# Concern about the possibility of becoming a victim of extortion: validation of a brief scale for Peruvian citizens

**DOI:** 10.3389/fpsyt.2025.1644797

**Published:** 2025-10-17

**Authors:** Oscar Mamani-Benito, Renzo Felipe Carranza Esteban, María Celinda Cruz Ordinola, Mariné Huayta-Meza, Cristhian Cruz-Campos, Milagros Yesenia Pacheco-Vizcarra, Wilter C. Morales-Garcia

**Affiliations:** ^1^ Facultad de Ciencias de la Salud, Universidad Señor de Sipán, Chiclayo, Peru; ^2^ Grupo de Investigación Avances en Investigación Psicológica, Facultad de Ciencias de la Salud, Universidad San Ignacio de Loyola, Lima, Peru; ^3^ Facultad de Ciencias Empresariales, Universidad Peruana Unión, Juliaca, Peru; ^4^ Facultad de Ciencias de la Salud, Universidad Peruana Unión, Juliaca, Peru; ^5^ Facultad de Ciencias Contables y Administrativas, Universidad Nacional del Altiplano, Puno, Peru; ^6^ Dirección General de Investigación, Universidad Peruana Unión, Lima, Peru

**Keywords:** extortion concern, psychometric validation, mental health, unidimensional scale, Peruvian population

## Abstract

**Introduction:**

The phenomenon of extortion is generating serious repercussions on the mental health of the economically active population. In the absence of measurement instruments to quantify the magnitude of this problem, it becomes urgent to design a documentary measurement tool.

**Objective:**

To design and validate a scale measuring concern about the possibility of becoming a victim of extortion.

**Method:**

The study is classified as instrumental. Using purposive non-probability sampling, participation was obtained from 2.049 citizens of both sexes across the three regions of Peru. The instrument was designed in 10 stages, following expert recommendations on the subject. The first version consisted of 11 items with five-point Likert-type response options. Analyses were conducted to demonstrate content validity, construct validity, convergent validity, measurement invariance, and reliability.

**Results:**

All items proved to be clear, relevant, and representative (V > 0.70). Exploratory factor analysis suggested an underlying structure composed of eight items (KMO = 0.91, Bartlett’s test = p ≤ 0.001), with factor loadings above the 0.40 cutoff (0.75 to 0.83). Subsequently, confirmatory factor analysis corroborated this unidimensional structure (SRMR = 0.037, RMSEA = 0.074, CFI = 0.996, TLI = 0.994). In addition, the BECS was shown to be invariant across sex and exhibited significant correlations with other comparable scales, thus providing evidence of convergent validity. Finally, the instrument demonstrated excellent reliability (>0.90).

**Conclusion:**

The BECS shows psychometric evidence supporting its validity and reliability. Therefore, it becomes the first measure available to assess concern about the possibility of becoming a victim of extortion.

## 1 Introduction

Extortion is one of the most persistent, structural, and least visible forms of criminal violence in Latin America, affecting not only local economies but also the institutional stability and social fabric of countries in the region región (1). This practice has become deeply rooted in territories characterized by high levels of informality, clientelist networks, institutional corruption, and fragile judicial systems (1–3). Consequently, it is not limited to formal economic sectors but instead establishes a pattern of victimization that exacerbates structural inequalities (4).

In countries such as Mexico, El Salvador, and Guatemala, extortion has ceased to be a marginal phenomenon and has evolved into a form of parallel criminal governance, where extortionists partially supplant the State by imposing their own logic of control and collection (1). The magnitude of underreporting of this crime is alarming. For instance, in Mexico, 97.9% of cases go unreported (5), while in Colombia the figure reaches 81.4% (6). This situation reflects not only the fear of reprisals but also the deep-seated structural mistrust in judicial institutions.

In the Peruvian context, recent data revealed that in 2024, 27.5% of the population over 15 years of age had been victims of some criminal act (7), with merchants identified as the occupational group most affected by extortion (8). Other reports highlight the negative impact on the mental health of the economically active population (7, 9), particularly among those working in informal sectors, which makes them even more vulnerable to extortion (10).

### 1.1 Literature review

Extortion as a crime has been the subject of research in various regions of the world. Studies highlight its prevalence and the psychological impact on victims (11). Although its modalities vary according to geographic and socioeconomic contexts, it is clearly a serious threat in countries where organized crime predominates and weaknesses in state control are evident (12). From a psychological perspective, the emotional consequences of extortion have been documented in several international, national, and local studies. For example, research conducted in Mexico with adolescents and young people living in environments marked by high levels of social disorder, vandalism, and crime revealed heightened psychological distress as a reaction (13). Another study with Latin American migrants who arrived at the U.S. border found that adults traveling with children were the most extorted, and when evaluated, they reported significant symptoms of post-traumatic stress (11). Similarly, in India, a study conducted with female students who were victims of electronic extortion revealed that the main mental health consequences included suicide attempts, fear of scandal, and frequent monitoring of social networks due to fear that the extortionist would disseminate the victim’s information (14). Finally, in Peru, research exploring the influence of “quota collections” and the impact of telephone extortion revealed serious alterations in the perception of well-being among transport workers, merchants, and public officials (15).

For an evidence-based understanding, these effects must be framed within a broader theoretical context in which extortion is defined as the imposition of payments under threat or intimidation (16, 17). Its prevalence is tied to a “booming industry” that thrives in contexts where strategies to curb organized crime and drug trafficking have failed due to weak state control (18). Within this framework, it is important to distinguish the different reactions the population may have toward this phenomenon. Fear of crime, for instance, is an emotional response encompassing feelings of vulnerability and insecurity in the face of a real threat (19). Direct victimization is the tangible experience of having suffered a criminal act, with emotional, social, and legal consequences (11). Meanwhile, concern about the possibility of becoming a victim of extortion based on emerging studies that show its relevance in understanding psychosocial responses to specific crimes (20) can be understood as a complex, multidimensional psychological state. First, it includes a cognitive dimension, involving recurrent thoughts about the risk of being victimized by this crime (21). Second, it entails an emotional impact manifested through indicators such as anxiety, nervousness, and emotional distress (22). Third, it is reflected in avoidance behaviors actions that individuals take to reduce their exposure to the risk of extortion (23). Finally, it involves perceived control, understood as the degree of confidence an individual has in facing extortion situations (12).

To understand this phenomenon, classic theoretical models such as the fear of crime theory suggest that not only individual factors but also social and contextual factors play a key role in explaining fear (24, 25). In the case of extortion victimization, from an integrative criminological approach, various theories reveal its multicausal nature, among which Rational Choice Theory (26) and Routine Activity Theory (27) stand out. Rational Choice Theory, in particular, explains why some victims comply with extortion demands while others resist. From this perspective, compliance depends on a subjective evaluation of costs and benefits (28, 29), where factors such as the credibility of the threat, the presence of weapons, the number of aggressors, or the type of communication (face-to-face or remote) are key determinants shaping the perception of risk and, therefore, the decision to pay (30, 31). Along the same line, another relevant model is Protection Motivation Theory (PMT), developed by Rogers (32), which explains how people evaluate and respond to perceived threats. In this case, the motivation to adopt protective behaviors depends on the perceived severity of the threat, personal vulnerability, and the perceived efficacy of the available responses (33). Applied to extortion, this model conceptualizes concern as a cognitive and emotional evaluation mechanism, reflecting perceptions of risk and vulnerability in the face of this specific threat (34).

Against this backdrop, it is essential to have measurement instruments to quantify the emotional impact generated by extortion on the Peruvian population, both in direct victims (11) and in those indirectly affected (35). Current literature offers a variety of tools to assess broader aspects, such as fear of crime, which includes extortion as one among many criminal acts. In the last 25 years (36), this phenomenon has been mainly assessed through surveys (77%), structured or semi-structured interviews (18%), and other methods involving secondary analyses, observation, and real-time apps (<5%). One of the most widely used international scales is the Fear of Crime Scale, created by Jackson (37) for adults in London, United Kingdom. This scale is composed of 16 items distributed across four dimensions: concern about crime, perceived probability of crime, perceived control over crime, and perceived consequences of crime. Later, adaptations were developed for young populations in Mexico (38) and Ecuador (39), in both cases confirming the original structure.

Other tools have also emerged, such as the one created by Grijalva-Eternod and Fernández-Molina (40), notable for its integral approach to cognitive, behavioral, and generalized concern aspects of fear of crime. This instrument was validated in Spain and comprised 9 items distributed across three dimensions: abstract fear, cognitive fear, and behavioral fear. Likewise, Valera and Guardia (41) constructed a test to evaluate perceived insecurity and fear of crime among residents of Barcelona, Spain. This instrument consisted of 45 items distributed across eight subscales: perception of insecurity, previous threat experiences, social representations of insecurity, personal control and coping skills, potential aggressors in public spaces, neighborhood urban identity, residential satisfaction, and perceived environmental quality.

Although most of the available measures are characterized by multidimensionality, other alternatives stand out for their brevity and unidimensionality. One such case is the scale developed by Etopio and Berthelot (42), who designed a tool for adults in the United States using in-depth interviews, resulting in a short 10-item, single-dimension measure. Another instrument is the Concern About Becoming a Victim of Robbery Scale, created by Carranza et al. (20) for Peruvian citizens, which consisted of 5 items distributed in a single factor.

### 1.2 Justification

Returning to the issue of interest, it is necessary to recognize that in order to understand and address the psychosocial consequences of extortion, not only scientific studies are required, but also public policies that integrate mental health strategies, crime prevention, and social support (43),.This has been emphasized by various experts and international organizations, who call on countries with high levels of citizen insecurity to promote community resilience and protect victims (44, 45). However, in the Peruvian context, the lack of specific instruments to measure concern and the effects of extortion limits the capacity to design and evaluate such policies. It is precisely this gap in knowledge that motivates the present study.

In this regard, since most instruments available in the scientific literature have been designed to evaluate fear of crime in a general sense, there is a clear need for specific scales aimed at assessing fear related to particular crimes, such as concern about extortion a topic that is still in the process of consolidation within academic research (46). Although some alternatives exist that may serve as a basis for potential adaptation and validation, the authors of this study consider it necessary to construct a test that directly measures the construct of interest, given that the tools identified in the literature are not directly aligned with the objectives of the present research.

### 1.3 Hypotheses

Based on the above, the following research hypotheses are proposed:

- The scale measuring concern about the possibility of becoming a victim of extortion demonstrates content validity, ensuring that its items adequately reflect the construct in the Peruvian context.
- The factorial structure of the scale fits a unidimensional model, confirming that the items consistently measure a single underlying dimension.
- The scale demonstrates measurement invariance across relevant subgroups, guaranteeing the validity of comparisons between these groups without bias in the structure or interpretation of the items.
- The scale shows convergent validity, as it correlates significantly with related constructs, such as measures assessing concern about robbery and anxiety.
- The scale demonstrates high internal consistency, ensuring reliability and precision of measurements in the Peruvian population.

### 1.4 Objective

For all the reasons mentioned, the objective of this research is to design and validate a scale measuring concern about the possibility of becoming a victim of extortion among Peruvian citizens.

## 2 Method

### 2.1 Design

The study is characterized as having an instrumental design (47), since it explores the main psychometric properties of a documentary measurement instrument.

### 2.2 Participants

The study population consisted of Peruvian citizens of both sexes, aged 18 years and older. The sample size was calculated using *semPower*, a package from the R software (48). A total of 55 degrees of freedom was considered, corresponding to the complexity of the factorial model with 11 items. In addition, rigorous statistical parameters were established: an RMSEA index of 0.05 to ensure good model fit, a significance level of 0.05 to control for Type I error, and a statistical power of 0.80 to minimize Type II error. Under these specifications, the calculation yielded a minimum sample size ranging from 180 to 220 participants, which guarantees reliable results for both exploratory and confirmatory factor analyses.

Due to limitations in accessibility to the study population, a purposive non-probability sampling method was selected. This approach ensured the voluntary participation of 2,049 individuals, from which 289 were excluded for not meeting the established inclusion and exclusion criteria. These criteria were defined prior to data collection and were based on the need to obtain a relevant, ethical sample with data of sufficient quality to adequately analyze the psychometric properties of the instrument. In this case, only participants over the age of 18 were considered, as the scale is intended for an adult population with the capacity to provide valid and complete informed consent. Likewise, only those who fully completed the questionnaire were included to guarantee the quality and completeness of the data. Conversely, individuals under the age of 18 were excluded to avoid ethical implications and differences in the experience of political stress related to age. Those who did not accept the informed consent were also excluded to comply with ethical standards, as well as individuals who did not complete the form, since their exclusion ensures the integrity of the psychometric analysis and minimizes biases derived from incomplete data.

After this filtering process, the final sample consisted of 1,760 citizens, of which more were women (58.6%) than men (41.4%). The age group with the highest participation was 18–30 years (84.4%). Regarding educational attainment, 59.7% reported higher education, 17.5% secondary education, and 1.5% primary education. In terms of region of residence, 53.1% lived in the highlands, 26.1% on the coast, and 20.7% in the jungle. Most participants were single (74.4%), followed by married or cohabiting (19.4%), divorced (3.3%), and widowed (2.9%). Finally, in terms of employment status, there was a higher proportion of salaried workers (54.5%) compared to self-employed/entrepreneurs (45.5%).

### 2.3 Instrument

The main scale was constructed following the recommendations of Muñiz and Fonseca-Pedrero (49). First, the theoretical framework was defined, and the justification for constructing the test was established. In this case, indicators were drawn from the theoretical model of fear of crime (39). Rational Choice Theory (26), and Routine Activity Theory (27).The justification was based on the absence of similar instruments that could even be considered for adaptation. Second, the construct was analyzed and defined, delimiting its conceptual boundaries and dimensions. As a result, the proposed factors were: Factor 1 (Cognitive Concern), Factor 2 (Emotional Impact), Factor 3 (Avoidance Behaviors), and Factor 4 (Perceived Control).

Third, the items were drafted based on the nine indicators that operationally represent the four proposed factors: frequency of concern and perception of vulnerability (Factor 1); anxiety in the face of physical threats, fear of information exposure, and social shame (Factor 2); modification of routines and digital self-censorship (Factor 3); trust in authorities and response capacity (Factor 4). This stage resulted in an initial version consisting of 11 items with 5 Likert-type response options: Never, Rarely, Sometimes, Frequently, and Always. Fourth, to validate the content of the initial test, a panel of six experts was convened (two clinical psychologists, two forensic psychologists specializing in criminology, one psychiatrist, and one criminal lawyer). Using a validation form, they assessed the clarity, relevance, and representativeness of the items. Finally, the subsequent stages involved administering the instrument to the study population and conducting the psychometric analysis.

Other instruments were also used to assess whether the EPre-VE scores converged favorably with scales measuring theoretically related constructs. First, the Concern About Becoming a Victim of Robbery Scale (EPre-RD) (20), which consists of 5 statements with 5 Likert-type response options (Never, Rarely, Sometimes, Frequently, and Always). This measure showed a reliability coefficient of 0.91 according to Omega, indicating high reliability. Second, the Generalized Anxiety Disorder Scale-2 (GAD-2) (50), a brief scale that assesses anxiety, composed of 2 questions with 4 Likert-type response options (Not at all, Several days, More than half the days, Nearly every day). In this case, the Omega coefficient indicated a reliability value of 0.81.

### 2.4 Procedures

Data were collected through an online survey, since in recent years this medium has become the most cost-effective and efficient alternative for scientific research (51). In this case, Google Forms was chosen because it is a free tool with an intuitive interface, which facilitates the management and analysis of responses thanks to its variety of question types such as multiple choice and open text. The form was divided into sections (1): informed consent, which emphasized voluntary participation and confidentiality of data (2); collection of sociodemographic data; and (3) the instrument items. The survey link was disseminated via social media platforms such as Facebook and WhatsApp, which make it possible to reach diverse populations (52). Facebook, in particular, allows for targeted advertising, increasing sampling effectiveness (53, 54). The survey remained open from April 10 to April 24, 2025, and the completion time ranged from 5 to 10 minutes.

### 2.5 Data analysis

The statistical analysis process was conducted in several phases. First, to assess the normality of item distribution and identify whether any items displayed skewness or inadequate variability, a preliminary analysis of skewness and kurtosis values was conducted, expecting scores not to exceed ±1.50 (55). This step ensures that the factorial structure, reliability, and validity of the instrument are based on data that meet basic statistical assumptions (56).

Second, prior to conducting exploratory factor analysis (EFA), data adequacy was evaluated using the Kaiser-Meyer-Olkin (KMO) index (> 0.70). To determine whether sufficient correlation existed among items to justify factor analysis, Bartlett’s test was also applied (p < 0.05). During the EFA stage, the aim was to identify the latent dimensions that group the items; thus, a four-factor model was specified. To determine the optimal number of factors, parallel analysis was used (57). To estimate factor loadings and improve model interpretability, the unweighted least squares estimation method with Promin rotation was applied (58). To ensure that only items contributing significantly to a factor were retained, items with loadings below 0.40 or factorial complexity were eliminated (57). This procedure was carried out with a subsample of 524 participants.

Third, to validate and confirm the structure previously identified through EFA, confirmatory factor analysis (CFA) was conducted within a structural equation modeling (SEM) framework using the Variance-adjusted Weighted Least Squares (WLSMV) estimator, which allows verification of whether the theoretical model fits the observed data (58). To evaluate model fit, the following indices were considered: Tucker-Lewis Index (TLI > 0.90), Comparative Fit Index (CFI > 0.90), and Root Mean Square Error of Approximation (RMSEA < 0.08), following the recommendations of Hu and Bentler (59). This ensured that the proposed factorial model adequately represented the relationships between items and latent factors. This procedure was performed with the remaining 1.236 participants.

Fourth, internal consistency was evaluated using the omega coefficient (ω), which provides a more precise estimate of reliability by considering the different loadings and specific errors of each item (60), unlike Cronbach’s alpha, which assumes all items contribute equally (61). Fifth, to determine whether the scale measured the construct uniformly across different demographic groups, factorial invariance analysis by sex was conducted, following the recommendations of Wu and Estabrook (62). In this process, progressive constraints were applied to parameters to verify equivalence of factor structures, factor loadings, and intercepts between sexes. Models were evaluated using the following cut-off criteria: ΔRMSEA < 0.015 and ΔCFI ≤ 0.010 (63). This strengthens the external validity and generalizability of the instrument.

Finally, convergent validity analysis was conducted to confirm that the scale was not measuring an isolated construct but was adequately connected with theoretically related variables (64), thereby strengthening its interpretation and utility in the psychometric context. Pearson’s correlation coefficient (r) was used to assess whether significant relationships existed between concern about becoming a victim of extortion, concern about becoming a victim of robbery when withdrawing money from an ATM or bank, and anxiety.

All analyses were performed using the R programming language within the RStudio environment. The packages *lavaan* (65) and *semPlot* (66) were employed to facilitate data organization and model estimation.

### 2.6 Ethical considerations

This research followed the recommendations of the Declaration of Helsinki, which outlines ethical principles for research involving human subjects, primarily the requirement of obtaining informed consent. In line with these principles, the study was approved by the Ethics Committee of Universidad Señor de Sipán (Code 289-CIEI).

## 3 Results

### 3.1 Sample characteristics

**Table 1** shows that the 1,760 participants were mostly men (58.6%), young adults between 18 and 30 years old (84.4%), with higher education (81%), residing mainly in the highlands (53.1%) and in urban areas (81.2%). Regarding marital status, single participants predominated (74.4%), and in terms of employment, more than half were dependent workers (54.5%), with the majority holding employee positions (68.7%). In terms of occupational sector, the “other” category stood out (49.5%), followed by construction, education, and health. To ensure the robustness of the psychometric analyses, the sample was randomly divided into two subsamples: EFA (n = 524) and CFA (n = 1.236). The results show that both subsamples maintained comparable proportions across key variables such as sex, age, educational level, and region of residence, thereby ensuring equivalence and representativeness of the groups.

**Table 1:** Sociodemographic characteristics of the total sample and subsamples.

Demographic variables	Total (1760)		EFA (524)		CFA (1236)	
	n	%	n	%	n	%
Sex						
Female	728	41.40%	223	42.60%	505	40.90%
Male	1032	58.60%	301	57.40%	731	59.10%
Age						
18 to 30 years	1485	84.40%	443	84.50%	1042	84.30%
31 to 40 years	152	8.60%	52	9.90%	100	8.10%
41 years and older	123	7.00%	29	5.50%	94	7.60%
Educational Level						
Primary	27	1.50%	7	1.30%	20	1.60%
Secondary	308	17.50%	99	18.90%	209	16.90%
Higher education	1425	81.00%	418	79.80%	1007	81.50%
Region of Residence						
Coast	460	26.10%	132	25.20%	328	26.50%
Highlands	935	53.10%	276	52.70%	659	53.30%
Jungle	365	20.70%	116	22.10%	249	20.10%
Area of Residence						
Rural	331	18.80%	92	17.60%	239	19.30%
Urban	1429	81.20%	432	82.40%	997	80.70%
Marital Status						
Single	1309	74.40%	400	76.30%	909	73.50%
Married or cohabiting	342	19.40%	91	17.40%	251	20.30%
Divorced	58	3.30%	16	3.10%	42	3.40%
Widowed	51	2.90%	17	3.20%	34	2.80%
Employment Sector						
Communications	36	2.00%	9	1.70%	27	2.20%
Construction	153	8.70%	42	8.00%	111	9.00%
Education	156	8.90%	38	7.30%	118	9.50%
Entertainment	27	1.50%	10	1.90%	17	1.40%
Hospitality	22	1.30%	8	1.50%	14	1.10%
Restaurants	61	3.50%	19	3.60%	42	3.40%
Health	126	7.20%	43	8.20%	83	6.70%
Security	134	7.60%	36	6.90%	98	7.90%
Financial services	67	3.80%	18	3.40%	49	4.00%
Transportation	80	4.50%	20	3.80%	60	4.90%
Tourism	26	1.50%	8	1.50%	18	1.50%
Other	872	49.50%	273	52.10%	599	48.50%
Type of Work						
Salaried (dependent)	959	54.50%	297	56.70%	662	53.60%
Self-employed (independent)	801	45.50%	227	43.30%	574	46.40%
Work Position						
Managerial	551	31.30%	180	34.40%	371	30.00%
Employee	1209	68.70%	344	65.60%	865	70.00%

*f*, frequency; %, Percentage.

### 3.2 Content validity

As shown in **Table 2**, Aiken’s V coefficients for item relevance, representativeness, and clarity were satisfactory (V ≥ 0.70) for all items except 10 and 11, which fell below the minimum acceptable threshold (V = 0.70), indicating they did not adequately represent the intended construct.

**Table 2:** Aiken’s V for evaluation of item relevance, representativeness, and clarity.

Items	Item (n = 6)				Representativeness (n = 6)				Clarity (n = 6)			
	M	SD	V	IC 95%	M	SD	V	IC 95%	M	SD	V	IC 95%
Item1	3.00	0.00	1.00	0.85-1.00	3.00	0.00	1.00	0.85-1.00	3.00	0.00	1.00	0.85-1.00
Item2	3.00	0.00	1.00	0.85-1.00	3.00	0.00	1.00	0.85-1.00	2.50	0.84	0.83	0.63-0.94
Item3	3.00	0.00	1.00	0.85-1.00	3.00	0.00	1.00	0.85-1.00	2.33	0.82	0.78	0.57-0.90
Item4	3.00	0.00	1.00	0.85-1.00	2.67	0.82	0.89	0.69-0.97	2.33	0.82	0.78	0.57-0.90
Item5	2.83	0.41	0.94	0.76-0.99	2.50	0.84	0.83	0.63-0.94	2.33	0.82	0.78	0.57-0.90
Item6	3.00	0.00	1.00	0.85-1.00	2.67	0.82	0.89	0.69-0.97	2.67	0.82	0.89	0.69-0.97
Item7	3.00	0.00	1.00	0.85-1.00	3.00	0.00	1.00	0.85-1.00	2.83	0.41	0.94	0.76-0.99
Item8	2.67	0.82	0.89	0.69-0.97	2.33	0.82	0.78	0.57-0.90	2.33	0.82	0.78	0.57-0.90
Item9	3.00	0.00	1.00	0.85-1.00	3.00	0.00	1.00	0.85-1.00	3.00	0.00	1.00	0.85-1.00
Item10	2.00	1.26	0.67	0.45-0.83	2.17	1.17	0.72	0.51-0.87	2.33	1.21	0.78	0.57-0.90
Item11	2.00	1.55	0.67	0.45-0.83	2.00	1.55	0.67	0.45-0.83	2.00	1.55	0.67	0.45-0.83

M, mean; SD, standard deviation; V, Aiken’s V; 95% CI, confidence interval for V.

### 3.3 Descriptive analysis and item correlation

Regarding the preliminary item analysis, **Table 3** shows that the means ranged from 2.35 to 3.17, suggesting that most items were below the scale’s midpoint. Standard deviation values ranged from 1.25 to 1.33, which is optimal for discrimination. Skewness scores ranged from –0.08 to 0.65, indicating symmetric distributions (± 1.50), and all kurtosis values were negative (–1.16 to –0.58), indicating platykurtic distributions. Finally, most of the correlations between the items were found to be significant and of moderate magnitude, estimated using a polychoric correlation matrix.

**Table 3:** Descriptive statistics and item correlation matrix.

Items	M	SD	S	K	i1	i2	i3	i4	i5	i6	i7	i8	i9	i10	i11
i1	2.94	1.28	0.12	-1.06	1										
i2	2.96	1.31	0.12	-1.05	0.62**	1									
i3	2.92	1.28	0.14	-1.03	0.62**	0.64**	1								
i4	2.76	1.30	0.25	-1.02	0.65**	0.62**	0.65	1							
i5	2.87	1.29	0.19	-1.01	0.60**	0.65**	0.66**	0.67**	1						
i6	3.17	1.33	-0.08	-1.16	0.55**	0.58**	0.55**	0.56**	0.53**	1					
i7	3.14	1.31	-0.06	-1.13	0.61**	0.56**	0.63**	0.59**	0.64**	0.67**	1				
i8	2.89	1.34	0.23	-1.08	0.47**	0.52**	0.55**	0.57**	0.54**	0.63**	0.54**	1			
i9	2.95	1.28	0.13	-1.01	0.53**	0.54**	0.51**	0.56**	0.52**	0.53**	0.55**	0.63**	1		
i10	2.35	1.25	0.65	-0.58	0.16*	0.19*	0.21*	0.20*	0.15*	0.19*	0.18*	0.22*	0.21*	1	
i11	2.69	1.29	0.31	-0.98	0.11*	0.08	0.08	0.12*	0.12*	0.09	0.12*	0.14*	0.14*	0.37**	1

M, Mean, SD, Standard Deviation, As, Skewness, K, Kurtosis.

**Correlations are significant at <.01.

*Correlations are significant at <.05.

### 3.4 Exploratory factor analysis

At the exploratory factor stage (**Table 4**), sampling adequacy was excellent (KMO = 0.91; Bartlett’s χ² = 2555.65, p ≤ 0.001), confirming that the correlations among items were sufficient to justify the application of factorial techniques. Regarding factor loadings, the EFA suggested an underlying structure composed of eight items, with loadings ranging from 0.75 to 0.83 and communalities (h²) between 0.57 and 0.67, indicating that 57% to 67% of the variance of each item was explained by the common factor. However, items i1, i10, and i11 were eliminated due to factor loadings below 0.40, low communalities, and weak correlations with other items. Finally, excellent reliability was found (ω > 0.90).

**Table 4:** Exploratory factor analysis.

Ítem (Español)	Item (English)	Factor loading	h²
2. El pensar que puedo ser víctima de extorsión afecta mi estado emocional	2. Thinking that I could become a victim of extortion affects my emotional state	0.80	0.65
3. El pensar que podría ser manipulado(a) o coaccionado(a) para que me dejen trabajar, me angustia emocionalmente	3. Thinking that I might be manipulated or coerced into letting me work causes me emotional distress	0.81	0.66
4. Siento que puedo ser víctima de extorsión en los próximos meses.	4. I feel that I could be a victim of extortion in the coming months.	0.82	0.67
5. Me siento nervioso/a ante la posibilidad de que me amenacen con violencia si no cumplo exigencias de los extorsionadores	5. I feel nervous at the possibility of being threatened with violence if I do not comply with extortionists’ demands	0.82	0.67
6. Me preocupa que usen mis datos personales (fotos, mensajes) para chantajearme.	6. I worry that my personal data (photos, messages) could be used to blackmail me.	0.79	0.63
7. Me preocupa que puedan difundir información íntima sobre mí y/o mi familia, si no accedo a las exigencias de los extorsionadores	7. I worry that they could release intimate information about me and/or my family if I do not comply with extortionists’ demands	0.82	0.66
8. Evito retirar dinero en efectivo de cajeros o bancos por miedo a ser extorsionado/a.	8. I avoid withdrawing cash from ATMs or banks for fear of being extorted.	0.78	0.6
9. Limito mi actividad en redes sociales para reducir el riesgo de extorsión cibernética.	9. I limit my social media activity to reduce the risk of cyber extortion.	0.75	0.57
Reliability			
McDonald’s omega (ω) = 0.91			

F1, Single factor; h2, Communalities.

### 3.5 Confirmatory factor analysis

**Figure 1:**
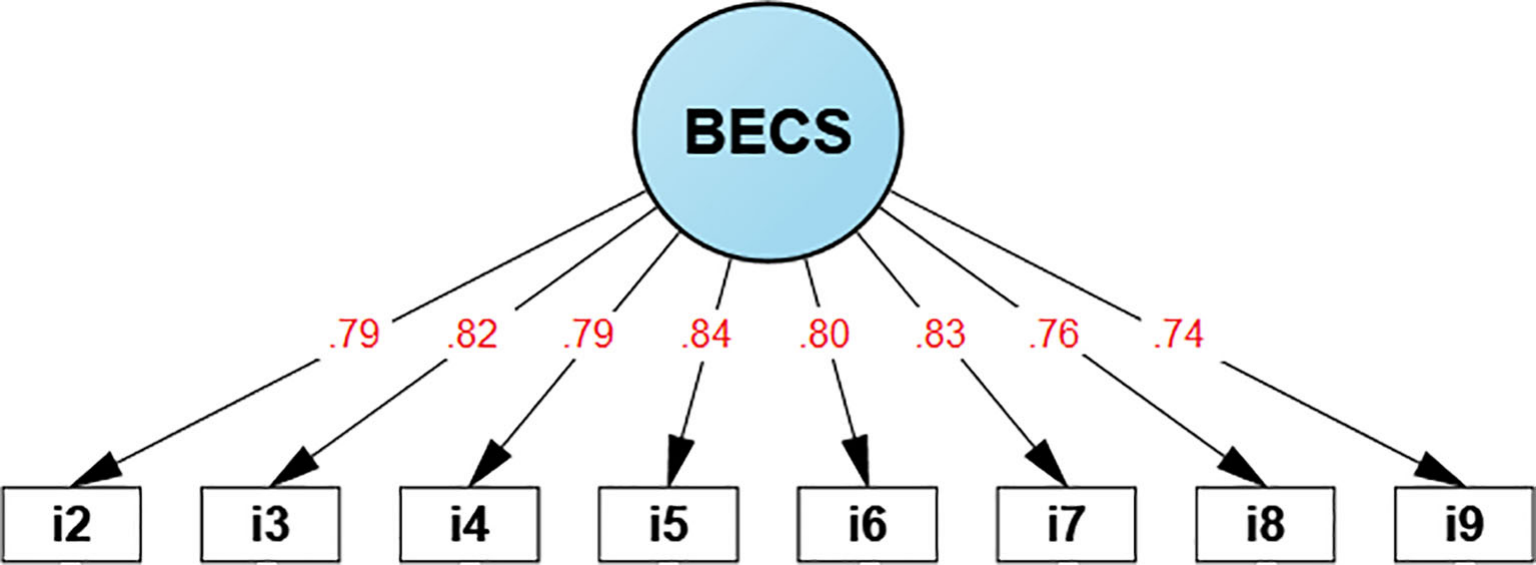
BECS internal structure.

The CFA results (**Table 5**) presents the main goodness-of-fit indices. First, the SRMR (0.037) and RMSEA (0.074) showed values below the 0.08 threshold. Similarly, the CFI and TLI reported values of 0.996 and 0.994, both exceeding the 0.95 benchmark. These findings confirm a favorable fit to the observed data, indicating that the 8-item version suggested by the EFA demonstrates validity based on internal structure. From this point onward, it will be referred to as the EPre-VE (see **Figure 1**).

**Table 5:** CFA goodness-of-fit indices.

Model	χ^2^	df	SRMR	CFI	TLI	RMSEA
Final	204.845	20	0.037	0.996	0.994	0.074

### 3.6 Measurement invariance

The analysis of measurement invariance, the results (**Table 6**) show that the EPre-VE is invariant across sex at all levels (configural, threshold, metric, scalar, and strict). In this case, the goodness-of-fit indices were adequate in all models, and the differences between them were minimal. This supports structural, metric, and scalar equivalence for both men and women; therefore, it can be affirmed that the EPre-VE measures the same construct equivalently across both groups.

**Table 6:** Measurement invariance by sex.

Model invariance	*χ* ^2^	gl	CFI	RMSEA	SRMR	ΔCFI	ΔRMSEA	ΔSRMR
Sex								
Configural	332.18	40	0.94	0.07	0.03	–	–	–
Threshold	332.18	39	0.94	0.07	0.35	0.000	0.002	0.316
Metric	339.13	46	0.94	0.06	0.03	0.000	-0.009	-0.312
Scalar	352.81	54	0.94	0.06	0.04	-0.001	-0.007	0.001
Strict	388.39	62	0.94	0.05	0.04	-0.005	-0.002	0.001

### 3.7 Validity based on relations with other variables

Finally, the correlation results support the convergent validity of the EPre-VE, as a strong and significant correlation was found with another similar measure, the Concern About Robbery Scale (r = 0.74, p < 0.01). Likewise, a moderate correlation was found with a related but distinct measure, the Generalized Anxiety Disorder Scale-2 (r = 0.36, p < 0.01).

## 4 Discussion

Extortion is a phenomenon that has a direct impact on the mental health and social well-being of the economically active population (1). The concern derived from this problem generates constant emotional insecurity, which represents a risk to public health (21). In this sense, addressing such concern proactively makes sense if preventive interventions are to be implemented (67). Therefore, given the lack of instruments to assess concern about the possibility of becoming a victim of extortion, the objective was to design and validate a scale for Peruvian citizens.

Overall, the findings of this study indicate that the EPre-VE should be interpreted as a brief, unidimensional measure consisting of eight items. Under this premise, the results of the preliminary analysis provide evidence of the consistency among items (significant correlations), which discriminate adequately and display relatively acceptable response distributions. However, this stage also revealed problematic items, such as i10 and i11, which showed low correlations and suggested the need for further revision in order to optimize the validity and reliability of the EPre-VE (68).

At the next level of analysis, although the initial construction of the scale proposed a four-factor structure, the EFA results indicated that all items loaded onto a single dimension. This means that, although the variable was theoretically conceived as multidimensional, empirically the data show that the items measure one underlying dimension (69). The authors interpret this reduction to a single factor as reflecting that the scale captures a coherent global construct (70), namely, concern about extortion as an integrated psychological experience rather than a fragmented phenomenon.

It is important to clarify that the EFA results only supported the unidimensionality of the eight-item scale. In this process, items i1 *“Lately I am concerned about the possibility of becoming a victim of extortion”*, i10 *“I trust that the police can protect me from extortion*”, and i11 “*I am clear on how I should act if I receive an extortion call or message*” were eliminated due to unfavorable statistical indicators, such as low factor loadings (below the recommended.40 in test construction), low communalities, and weak correlations. Conceptually and operationally, these items appear to measure aspects different from the main construct. Therefore, their removal was based on psychometric criteria recommended for optimizing the validity and reliability of psychological scales (71).

Subsequently, the CFA corroborated the validity of the underlying structure, confirming that the unidimensional model fits the observed data well. This consistency between the EFA and CFA demonstrates that the construction and validation process was successful, producing an instrument that is structurally valid (72) and, on the other hand, reliable, as the scale’s reliability reached an excellent value (>.90). This indicates that the scores obtained are stable and precise (73).

These findings support the notion that concern about becoming a victim of extortion, rather than being a fragmented phenomenon, represents an integrated psychological experience consistent with Rogers’ Protection Motivation Theory (PMT) (32). This theory posits that responses to specific threats are based on a unified cognitive and emotional evaluation of severity, vulnerability, and response efficacy (33, 34). Furthermore, this integration aligns with the Fear of Crime Theory, which conceptualizes these emotional responses as part of a global process involving individual, social, and contextual factors (24, 25).

In contrast to similar studies, our findings differ from related multidimensional scales, such as the cyberbullying victimization scale (three factors) (74) or the fear of crime assessment, which reports five-factor structures (39). These structures reflect the complexity of constructs that measure multiple facets of victimization or fear. Conversely, the BECS focuses exclusively on a single construct, favoring a unifactorial representation (75). This perspective is consistent with scales measuring fear of crime or concern about robbery, which exhibit unidimensional structures (20, 42). This suggests that, in contexts of targeted threats, integrated emotional and cognitive dimensions predominate as an indivisible whole (39, 76). Similarly, Rational Choice Theory (26–31) complements this interpretation by proposing that the subjective evaluation of costs and benefits when facing the threat of extortion functions as an integrated decision-making process, where factors such as threat credibility and operational context influence the unified perception of risk.

It is also worth noting that these results are more representative of a sample largely composed of young people (84.4%), women (58.6%), and individuals with higher education (59.7%), mainly from the highlands region (53.1%). Therefore, it must be acknowledged that perceptions of extortion may vary according to cultural and regional differences, particularly when contrasting urban and rural contexts or different occupational groups (77). These variations can influence how individuals experience and express concern about extortion, as social, economic, and cultural factors specific to each context shape perceptions of risk and coping strategies (78).

Beyond the main findings, other results add value to the evidence of validity and reliability. First, measurement invariance showed that the EPre-VE is invariant by sex. This means that it measures concern about becoming a victim of extortion equivalently in men and women, thereby strengthening internal validity and the interpretation of scores (79), especially in research or interventions requiring group comparisons by sex (80, 81). Second, the correlations between the GAD-2 and the EPre-VE were significant, indicating that greater concern about becoming a victim of crime is associated with higher levels of generalized anxiety symptoms (82), which is consistent with psychological literature (83). Furthermore, the strong correlation between the two specific concerns (EPre-VE and EPre-RD) reinforces the idea that those with greater concern about extortion also tend to worry about robbery (84), which is reasonable given that both variables reflect concerns related to victimization and personal insecurity (85).

### 4.1 Practical implications

These findings support the notion that concern about becoming a victim of extortion, rather than being a fragmented phenomenon, represents an integrated psychological experience consistent with Rogers’ Protection Motivation Theory (PMT) (32). This theory posits that responses to specific threats are based on a unified cognitive and emotional evaluation of severity, vulnerability, and response efficacy (33, 34). Furthermore, this integration aligns with the Fear of Crime Theory, which conceptualizes these emotional responses as part of a global process involving individual, social, and contextual factors (24, 25).

In contrast to similar studies, our findings differ from related multidimensional scales, such as the cyberbullying victimization scale (three factors) (74) or the fear of crime assessment, which reports five-factor structures (39). These structures reflect the complexity of constructs that measure multiple facets of victimization or fear. Conversely, the BECS focuses exclusively on a single construct, favoring a unifactorial representation (75). This perspective is consistent with scales measuring fear of crime or concern about robbery, which exhibit unidimensional structures (20, 42). This suggests that, in contexts of targeted threats, integrated emotional and cognitive dimensions predominate as an indivisible whole (39, 76). Similarly, Rational Choice Theory (26–31) complements this interpretation by proposing that the subjective evaluation of costs and benefits when facing the threat of extortion functions as an integrated decision-making process, where factors such as threat credibility and operational context influence the unified perception of risk.

### 4.2 Limitations

First, although the sample is large and includes participants from the three regions of Peru, it is not representative of the sociocultural and demographic diversity of this population. Combined with the lack of randomness due to the use of non-probability sampling, this limits the generalizability of the findings to the entire Peruvian population. Second, since the data were collected through self-report instruments, there is a possibility of social desirability bias, as a sensitive topic like concern about extortion may have affected the accuracy of responses. Third, not considering potentially moderating variables such as socioeconomic factors or previous victimization experiences may have influenced the intensity of the measured concern, thus limiting the comprehensive understanding of the phenomenon. Fourth, this scale primarily measures emotional responses and perceived vulnerability to extortion, which are key to understanding the initial psychological impact of the phenomenon. However, it does not address cognitive and behavioral dimensions or self-efficacy in resisting threats, which are fundamental elements for coping. Therefore, interpretation of the results should be limited to the affective dimension of concern. Five, this study did not include additional behavioral or emotional variables with which the predictive validity of the scale could be tested. Lastly, another important limitation is that test-retest reliability was not evaluated, which prevents establishing the temporal stability and consistency of the BECS over time.

In light of these limitations, it is recommended that future research apply probabilistic sampling techniques with more diverse samples to improve generalizability. Among the recommended techniques are simple random sampling or stratified sampling, which ensure randomness and proportional representativeness of subgroups, thereby minimizing selection bias. It is also advised to reduce social desirability bias by guaranteeing anonymity and using qualitative methods, such as semi-structured interviews or focus groups, which facilitate a deeper understanding of the phenomenon and validate the interpretation of the data. Additionally, it is important to include contextual and demographic variables, such as socioeconomic factors and previous experiences of victimization, to better understand concern about extortion. From a psychometric perspective, the scale should be expanded to incorporate cognitive, behavioral, and emotional indicators, such as avoidance, distress, or perceived insecurity, in order to assess its predictive and discriminant capacity. Furthermore, it is essential to examine whether the scale adequately distinguishes between concerns specifically related to extortion and other types of fear or stress, particularly in environments with high crime incidence, to enhance its usefulness in applied contexts. Finally, future studies should include temporal stability analyses to strengthen the validity and practical applicability of the scale.

## 5 Conclusion

The lack of specific instruments to measure concern about extortion in the Peruvian context positions this study as a timely response to a national research priority. The factorial analyses carried out, together with evidence of measurement invariance, significant correlations with related scales, and the high reliability observed, support that the BECS demonstrates favorable psychometric performance in the Peruvian population. In this sense, the BECS emerges as a brief and effective tool to assess one of the many mental health alterations caused by the phenomenon of extortion. Due to its brevity and precision, this measure becomes an ideal tool in contexts where extortion is a growing phenomenon, characterized by its profound psychological impact on victims, as evidenced by recent studies reporting an increase in this crime and its underreporting due to fear of denunciation.

Incorporating the BECS into interventions and policies to safeguard the country’s public health could generate faster responses to the psychological needs of victims, thereby strengthening institutional capacity to address them. In sum, this measure fills a gap in the scientific literature, which until now had focused primarily on the legal and criminal aspects of extortion, contributing instead a psychological perspective on its psychosocial consequences and facilitating the design of more effective cross-sector strategies for the prevention and management of this growing phenomenon.

## Data Availability

The raw data supporting the conclusions of this article will be made available by the authors, without undue reservation.

## References

[1] Dammert L. Extortion: the backbone of criminal activity in Latin America. In: Jack D, editor. Gordon Institute for Public Policy, Florida International University, Miami (2021). Available online at: https://digitalcommons.fiu.edu/jgi_research/47 (Accessed May 9, 2025).

[2] Garzón VJC. From Drug Cartels to Predatory Micro Networks: the “new” face of organized crime in Latin America. In: Bagley BM Rosen JD Kassab HS, editors. Reconceptualizing Security in the Western Hemisphere in the 21st Century. Maryland, EE.UU: Lexington Books (2014). p. 117–29. Available online at: https://www.academia.edu/17053036/From_Drug_Cartels_to_Predatory_Micro_Networks_the_new_face_of_organized_crime_in_Latin_America (Accessed May 9, 2025).

[3] Scaglione A. Cosa Nostra and Camorra: illegal activities and organisational structures. Glob Crime. (2016) 17:60–78. doi: 10.1080/17440572.2015.1114919

[4] Bonello D. Mujeres de Guatemala: ¿La nueva cara de la extorsión? InSight Crime (2019). Available online at: https://insightcrime.org/es/investigaciones/mujeres-de-Guatemala-la-nueva-cara-de-la-extorsion-2/ (Accessed May 9, 2025).

[5] Instituto Nacional de Estadística y Geografía (INEGI). Encuesta Nacional de Victimización y Percepción sobre Seguridad Pública (ENVIPE) 2014: Principales Resultados. México City: INEGI (2014). Available online at: https://www.inegi.org.mx/programas/envipe/2014/ (Accessed May 9, 2025).

[6] Departamento Administrativo Nacional de Estadística (DANE). Boletín Técnico Encuesta de Convivencia y Seguridad Ciudadana. Bogotá: DANE (2021). Available online at: https://www.dane.gov.co/files/investigaciones/poblacion/convivencia/2019/Bol_ECSC_2019.pdf (Accessed May 9, 2025).

[7] Silvestre CLR Guivar ZD Morales ORF. Causas y efectos de la criminalidad organizada en el Perú: Análisis integral. Rev Acad Esc postgrado la policía Nac Perú. (2025) 4:1–17. doi: 10.59956/escpograpnpv4n2.1

[8] Policia Nacional del Perú (PNP). Boletín informativo 2025. Lima: Gobierno del Perú (2025). Available online at: https://cdn.www.gob.pe/uploads/document/file/7942663/6679000-boletin-informativo-2025.pdf (Accessed May 11, 2025).

[9] Reyes VAC. Tendencia de la criminalidad organizada en el Perú: enfocada en al trata de personas. Rev Cienc e Investig en Def. (2025) 1:29–57. Available online at: https://recide.caen.edu.pe/index.php/recide/article/view/198.

[10] Instituto Nacional de Estadística e Informática (INEI). Producción y empleo informal en el Perú. Lima: INEI (2024). Available online at: https://www.gob.pe/inei/ (Accessed May 11, 2025).

[11] Vargas L Epperson CN Richmond TS Sharif S Berkowitz L Giano Z. Extortion experiences of recent adult immigrants from Latin America: self-reported prevalence, associated costs, and current mental health. Inj Epidemiol. (2024) 11:1–8. doi: 10.1186/s40621-024-00524-2, PMID: 39238066 PMC11376018

[12] Estévez-Soto PR. Determinants of extortion compliance: Empirical evidence from a victimization survey. Br J Criminol. (2021) 61:1187–205. doi: 10.1093/bjc/azab007

[13] Pérez-Sastré MA García-Peña C Ramos-Lira L Ortiz-Hernández L. Beyond direct exposure to violence: effects of living in disordered and violent communities on psychological distress in young Mexican people. Cad Saude Publica. (2024) 40:e00058123. doi: 10.1590/0102-311XEN058123, PMID: 38324861 PMC10841348

[14] Kareem AF. Electronic extortion and its impact on university female students. Univ Muhammadiyah Sidoarjo Indones J Heal Sci Med. (2025) 2:1–16. doi: 10.21070/ijhsm.v2i1.151

[15] Cajamarca CC Suarez PH. Impacto del delito de extorsión en la seguridad ciudadana en un distrito de Perú- San Martín de Porres. Regunt. (2025) 5:59–68. doi: 10.18050/regunt.v5i1.06

[16] Elsenbroich C Badham J. The extortion relationship: A computational analysis. Jasss. (2016) 19:1. doi: 10.18564/jasss.3223

[17] Savona EU Sarno F. Racketeering. In: Encyclopedia of Criminology and Criminal Justice. Springer Nature, New York (2014). p. 4264–73. doi: 10.1007/978-1-4614-5690-2_633

[18] Aburto JM Riffe T Canudas-Romo V. Trends in avoidable mortality over the life course in Mexico, 1990-2015: A cross-sectional demographic analysis. BMJ Open. (2018) 8:e022350. doi: 10.1136/bmjopen-2018-022350, PMID: 30068622 PMC6074636

[19] Silva C Guedes I. The role of the media in the fear of crime: A qualitative study in the Portuguese context. Crim Justice Rev. (2023) 48:300–17. doi: 10.1177/07340168221088570

[20] Carranza ERF Mamani-Benito OJ Turpo CJE Vilafuerte D la CA Elguera PA Lingan SK. Design and validation of a scale of concern about being a victim of robbery when withdrawing money from an ATM or bank (EPre-RD) in Peruvian citizens. J Police Crim Psychol. (2024). doi: 10.1007/s11896-024-09662-1

[21] Lantz L Chang Z Virtanen S. Risk factors for dropout from psychological substance use disorder treatment programs in criminal justice settings. Drug Alcohol Depend. (2024) 259:111314. doi: 10.1016/j.drugalcdep.2024.111314, PMID: 38696932

[22] Mugambiwa S Rakubu K. Shadows of fear: Extortion and protection racketeering amidst organized crime in South Africa. Edelweiss Appl Sci Technol. (2024) 8:2122–9. doi: 10.55214/25768484.v8i4.1586

[23] Obeid S Bitar Z Malaeb D Sakr F Dabbous M Hallit S. Psychometric properties of the Feeling of Unsafety Scale-Arabic in general population adults. Front Public Heal. (2025) 13:1491691. doi: 10.3389/fpubh.2025.1491691, PMID: 40226324 PMC11985478

[24] Gabriel U Greve W. The psychology of fear of crime. Conceptual and methodological perspectives. Br J Criminol. (2003) 43:600–14. doi: 10.1093/bjc/43.3.600

[25] Hale C. Fear of crime: A review of the literature. Int Rev Victimol. (1996) 4:79–150. doi: 10.1177/026975809600400201

[26] Abbasi H. Study of victimization؛Causation and explanation about efficiency of codes of the elderlies in Iranian criminal law. Int J Adv Stud Humanit Soc Sci. (2013) 2:108–17. Available online at: https://www.magiran.com/p2646786.

[27] Cohen LE Felson M. Social Change and crime rate trends: a routine activity approach. Am Sociol Rev. (1979) 44:588–608. Available online at: https://www.jstor.org/stable/2094589 (Accessed May 24, 2025).

[28] Konrad BK Skaperdas S. Extortion. Economica. (1998) 65:461–77. doi: 10.1111/1468-0335.00141

[29] Smith A Varese F. Payment, protection and punishment: the role of information and reputation in the mafia. Ration Soc. (2001) 13:357–93. doi: 10.1177/104346301773166954

[30] Estévez-Soto PR Johnson SD Tilley N. Are repeatedly extorted businesses different? A multilevel hurdle model of extortion victimization. J Quant Criminol. (2021) 37:1115–57. doi: 10.1007/s10940-020-09480-8

[31] Rockmann KW Northcraft GB. To be or not to be trusted: The influence of media richness on defection and deception. Organ Behav Hum Decis Process. (2008) 107:106–22. doi: 10.1016/j.obhdp.2008.02.002

[32] Rogers RW. A protection motivation theory of fear appeals and attitude change1. J Psychol. (1975) 91:93–114. doi: 10.1080/00223980.1975.9915803, PMID: 28136248

[33] Marikyan D Papagiannidis S. Protection Motivation Theory: a review. In: Papagiannidis S (Ed.), TheoryHub Book: this handbook is based on the online theory resource: TheoryHub. Newcastle upon Tyne: Newcastle University (2023). p. 78–93. Available online at: https://open.ncl.ac.uk/theory-library/TheoryHubBook.pdf (Accessed May 28, 2025).

[34] Crossler RE. Protection Motivation Theory: Understanding the Determinants of Individual Security Behavior. Blacksburg (VA): Virginia Polytechnic Institute and State University (2009). Available online at: https://vtechworks.lib.vt.edu/server/api/core/bitstreams/847c5b4c-fdce-4c62-9d47-5c11b2ca6add/content (Accessed May 26, 2025).

[35] Balmori-de-la-Miyar J Tennyson S Silverio-Murillo A Pagán JA. Violent crime victimization and mental health among adolescents in Mexico. Prev Med Rep. (2025) 53:103062. doi: 10.1016/j.pmedr.2025.103062, PMID: 40270917 PMC12017911

[36] Hart TC Chataway M Mellberg J. Measuring fear of crime during the past 25 years: A systematic quantitative literature review. J Crim Justice. (2022) 82:101988. doi: 10.1016/j.jcrimjus.2022.101988

[37] Jackson J. A psychological perspective on vulnerability in the fear of crime. Psychol Crime Law. (2009) 15:365–90. doi: 10.1080/10683160802275797

[38] Reyes Sosa H Álvarez Montero F Puente Martínez A. Miedo al delito en jóvenes mexicanos: propiedades psicométricas de una medida psicosocial. Pensando Psicol. (2020) 16:1–19. doi: 10.16925/2382-3984.2020.01.02

[39] Reyes-Sosa H Molina-Coloma V. Psychometric analysis of a scale to measure fear of crime in Ecuadorian youths. Acta Colomb Psicol. (2018) 21:300–9. doi: 10.14718/ACP.2018.21.1.13

[40] Grijalva-Eternod ÁE Fernández-Molina E. La multidimensionalidad del miedo al delito. Propuesta y validación de una escala para su medición. Política Crim. (2021) 16:497–523. doi: 10.4067/s0718-33992021000200497

[41] Valera S Guàrdia J. Perceived insecurity and fear of crime in a city with low-crime rates. J Environ Psychol. (2014) 38:195–205. doi: 10.1016/j.jenvp.2014.02.002

[42] Etopio AL Berthelot ER. Defining and measuring fear of crime: A new validated scale created from emotion theory, qualitative interviews, and factor analyses. Criminol Crim Justice Law Soc. (2022) 23:46–67. doi: 10.54555/ccjls.4234.34104

[43] Ministerio de Justicia y Derechos Humanos. Dinero y amenaza: proceso, modalidades y estructuras de la extorsión en el Perú. Lima: Ministerio de Justicia y Derechos Humanos (2025). Available online at: https://cdn.www.gob.pe/uploads/document/file/8163777/6833195-dinero-y-amenza_proceso-modalidades-y-estructura-de-la-extorsion-en-el-peru_03-de-junio.pdf?v=1749045926 (Accessed May 20, 2025).

[44] Gaitán D Noriega L Sequeira L. Manual de acción para la resiliencia comunitaria contra la extorsión en Centroamérica. Ginebra: Global Initiative Against Tansnational Organized Crime (2024). Available online at: https://globalinitiative.net/wp-content/uploads/2024/05/GI-TOC-Manual-de-Acción-2024-Web.pdf (Accessed May 20, 2025).

[45] Ramírez Irías L. Resiliencia comunitaria frente a la extorsión: Experiencias de El Salvador, Guatemala y Honduras. Ginebra: Global Initiative Against Tansnational Organized Crime (2024). Available online at: https://reliefweb.int/report/el-salvador/resiliencia-comunitaria-contra-la-extorsion-experiencias-de-el-salvador-Guatemala-y-Honduras-julio-2024 (Accessed May 20, 2025).

[46] Brands J Jansen JM van Doorn J Spithoven R. Measuring and explaining situational fear of crime: an experimental study into the effects of disorder, using virtual reality and multimodal measurement. Br J Criminol. (2025) 65:673–90. doi: 10.1093/bjc/azae072

[47] Ato M López JJ Benavente A. A classification system for research designs in psychology. Psicol. (2013) 29:1038–59. doi: 10.6018/analesps.29.3.178511

[48] Quispe AM Pinto DF Huamán MR Bueno GM Valle-Campos A. Metodologías cuantitativas: Cálculo del tamaño de muestra con STATA y R. Rev Cuerpo Médico HNAAA. (2020) 13:78–83. doi: 10.35434/rcmhnaaa.2020.131.627

[49] Muñiz J Fonseca-Pedrero E. Diez pasos para la construcción de un test. Psicothema. (2019) 31:7–16. doi: 10.7334/psicothema2018.291, PMID: 30664405

[50] Baños-Chaparro J. Análisis psicométrico del generalized anxiety disorder-2 en adultos Peruanos. Rev Investig Univ del Quindío. (2022) 34:30–6. doi: 10.33975/riuq.vol34n1.581

[51] Regmi PR Waithaka E Paudyal A Simkhada P Van Teijlingen E. Guide to the design and application of online questionnaire surveys. Nepal J Epidemiol. (2016) 6:640–4. doi: 10.1080/10705511.2014.950896, PMID: 28804676 PMC5506389

[52] Sloan L Quan-Haase A Salmons J. Using social media in data collection: designing studies with the qualitative E-research framework. In: The SAGE Handbook of Social Media Research Methods. SAGE Publications, London (2022). p. 123–45. doi: 10.4135/9781473983847.n12

[53] Franz D Marsh HE Chen JI Teo AR. Using facebook for qualitative research: A brief primer. J Med Internet Res. (2019) 21:e13544. doi: 10.2196/13544, PMID: 31411143 PMC6711038

[54] Schneider D Harknett K. What’s to like? Facebook as a tool for survey data collection. Sociol Methods Res. (2022) 51:108–40. doi: 10.1177/0049124119882477, PMID: 36845408 PMC9957582

[55] Kline RB. Principles and Practice of Structural Equation Modeling. 5th. New York: Guilford Publications (2023). Available online at: https://www.guilford.com/books/Principles-and-Practice-of-Structural-Equation-Modeling/Rex-Kline/9781462551910 (Accessed May 20, 2025).

[56] Tabachnick BG Fidell LS. Using Multivariate Statistics. 7th. Boston: Pearson (2019).

[57] Lloret-Segura S Ferreres-Traver A Hernández-Baeza A Tomás-Marco I. El análisis factorial exploratorio de los ítems: una guía práctica, revisada y actualizada. Psicol. (2014) 30:1151–69. doi: 10.6018/analesps.30.3.199361

[58] Méndez A. El análisis factorial: una introducción conceptual para la enseñanza y aprendizaje. Enseñanza e Investig en Psicol Nueva Época. (2024) 6:1–13. doi: 10.62364/cneip.6.1.2024.240

[59] Brown TA. Confirmatory factor analysis for applied research. 2nd. New York: Guilford Press (2015).

[60] Hu L Bentler PM. Cutoff criteria for fit indexes in covariance structure analysis: conventional criteria versus new alternatives. Struct Equ Modeling. (1999) 6:1–55. doi: 10.1080/10705519909540118

[61] Ventura-León J Caycho-Rodriguez T. El coeficiente Omega: un método alternativo para la estimación de la confiabilidad [Letter to the editor. Rev Latinoam Cienc Soc Niñez Juv. (2017) 15:625–7. Available online at: https://www.redalyc.org/pdf/773/77349627039.pdf (Accessed May 20, 2025).

[62] Sijtsma K. On the use, the misuse, and the very limited usefulness of cronbach’s alpha. Psychometrika. (2009) 74:107–20. doi: 10.1007/s11336-008-9101-0, PMID: 20037639 PMC2792363

[63] Wu H Estabrook R. Identification of confirmatory factor analysis models of different levels of invariance for ordered categorical outcomes. Psychometrika. (2016) 81:1014–45. doi: 10.1007/s11336-016-9506-0, PMID: 27402166 PMC5458787

[64] Finch H French B. Educational and Psychological Measurement. New York: Routledge (2018). doi: 10.4324/9781315650951

[65] Acuña I Michelini Y Guzmán JI Godoy JC. Assessment of convergent and discriminant validity in computerized decision-making tests. Aval Psicol. (2017) 16:375–83. doi: 10.15689/ap.2017.1603.12952

[66] Rosseel Y. Lavaan: An R package for structural equation modeling and more. J Stat Software. (2012) 48:1–36. Available online at: https://www.jstatsoft.org/article/view/v048i02 (Accessed May 22, 2025).

[67] Epskamp S. semPlot: Unified visualizations of structural equation models. Struct Equ Model Multidiscip J. (2015) 22:474–83. doi: 10.1080/10705511.2014.937847

[68] Ministerio del Interior. Política Nacional Multisectorial de Seguridad Ciudadana al 2030. Lima: Ministerio del Interior (2021). Available online at: https://cdn.www.gob.pe/uploads/document/file/3290003/PoliticaNacionalMultisectorial-SeguridadCiudadana2030.pdf?v=1656015453 (Accessed May 22, 2025).

[69] DeVellis RF. Scale Development: Theory and Applications. 4th. Los Angeles: SAGE Publications (2017).

[70] Borsboom D Mellenbergh GJ Van Heerden J. The theoretical status of latent variables. Psychol Rev. (2003) 110:203–19. doi: 10.1037/0033-295X.110.2.203, PMID: 12747522

[71] Reise SP Bonifay WE Haviland MG. Scoring and modeling psychological measures in the presence of multidimensionality. J Pers Assess. (2013) 95:129–40. doi: 10.1080/00223891.2012.725437, PMID: 23030794

[72] Ferrando PJ Lorenzo-Seva U Hernández-Dorado A Muñiz J. Decalogue for the factor analysis of test items. Psicothema. (2022) 34:7–17. doi: 10.7334/psicothema2021.456, PMID: 35048890

[73] Marsh HW Morin AJS Parker PD Kaur G. Exploratory structural equation modeling: An integration of the best features of exploratory and confirmatory factor analysis. Annu Rev Clin Psychol. (2014) 10:85–110. doi: 10.1146/annurev-clinpsy-032813-153700, PMID: 24313568

[74] Deng L Chan W. Testing the difference between reliability coefficients alpha and omega. Educ Psychol Meas. (2017) 77:185–203. doi: 10.1177/0013164416658325, PMID: 29795909 PMC5965544

[75] Chun JS Kim J Lee S. Development of a cyberbullying victimization scale for adolescents in South Korea. Child Youth Serv Rev. (2023) 144:106744. doi: 10.1016/j.childyouth.2022.106744

[76] Chen Q Lu Z Liu B Xiao Q Zhu Y CHan KL. Validation of the Chinese version of the coping strategies for victims of cyberbullying scale. BMC Psychol. (2024) 12:1–11. doi: 10.1186/s40359-024-01766-x, PMID: 38725028 PMC11084028

[77] Liu X Cao P Lai X Wen J Yang Y. Assessing essential unidimensionality of scales and structural coefficient bias. Educ Psychol Meas. (2023) 83:28–47. doi: 10.1177/00131644221075580, PMID: 36601253 PMC9806515

[78] Carranza-Esteban RF Mamani-Benito OJ Corrales-Reyes IE Landa-Barzola M Marca-Dueñas G Tito–Betancur VS. Psychometric evidence of a scale measuring concern over contagion with COVID-19 among Peruvian interns. Rev Cuba Investig Biomed. (2021) 40:e1289. Available online at: http://scielo.sld.cu/scielo.php?script=sci_arttext&pid=S0864-03002021000200008 (Accessed May 22, 2025).

[79] Laboratorio de Soluciones Colaborativas de Políticas Públicas (LABCO). ¿Cómo combatir la extorsión en el Perú? Lima: LABCO (2023). Available online at: https://propuestasdelbicentenario.pe/wp-content/uploads/2023/12/Como-combatir-la-extorsion-en-el-Peru.pdf (Accessed May 22, 2025).

[80] Pérez NC Galiano MG Vera ESB Rodríguez HDM. Cultura de la violencia: un análisis de las conexiones sociales y sus implicaciones en la delincuencia. Rev Cienc Tecnol Innov. (2023) 10:523–42. doi: 10.61154/rue.v10i4.3268

[81] Cheung GW Rensvold RB. Structural equation modeling: A evaluating goodness-of- fit indexes for testing measurement invariance. Struct Equ Model A Multidiscip J. (2009) 9:233–55. doi: 10.1207/S15328007SEM0902_5

[82] Valencia LPA Nateras GME Pacheco AM. Gender factors of objective and subjective insecurity: the cases of Mexico and Colombia. Polit Crim. (2023) 18:378–414. doi: 10.4067/S0718-33992023000100378

[83] Grijalva EAE Fernández ME. Diferencias de género en relación con el miedo al delito análisis en México. Univ Complitense Madrid. (2014) 2:432–43. Available online at: https://dialnet.unirioja.es/servlet/articulo?codigo=7286020 (Accessed May 28, 2025).

[84] Gutiérrez QJR Portillo GCB. La violencia delincuencial asociada a la salud mental en la población salvadoreña. Rev Psicol. (2014) 32:3–37. doi: 10.18800/psico.201401.001

[85] American Psychiatric Association. Más allá de la preocupación. Washington, DC: American Psychological Association (2017). Available online at: https://www.apa.org/topics/anxiety/preocupacion (Accessed May 28, 2025).

